# Smart Hydrogel Based on Derivatives of Natural α-Amino Acids for Efficient Removal of Metal Ions from Wastewater

**DOI:** 10.3390/gels10090560

**Published:** 2024-08-29

**Authors:** Monika Adamowska, Klaudia Kaniewska, Magdalena Muszyńska, Jan Romański, Wojciech Hyk, Marcin Karbarz

**Affiliations:** 1 Faculty of Chemistry, University of Warsaw, 1 Pasteura, PL 02-093 Warsaw, Poland; m.adamowska2@student.uw.edu.pl (M.A.); kkaniewska@chem.uw.edu.pl (K.K.); magdalena.muszynska@pepolska.pl (M.M.); jarom@chem.uw.edu.pl (J.R.); 2 Biological and Chemical Research Center, University of Warsaw, 101 Żwirki i Wigury Av., PL 02-089 Warsaw, Poland

**Keywords:** hydrogel, smart gel, amino acids, heavy metal ions, sorption properties

## Abstract

A novel class of hydrogels, rich in a variety of functional groups capable of interacting/complexing with metal ions was successfully synthesized. This was achieved by using acryloyl derivatives of natural α-amino acids, specifically ornithine and cystine. The δ-amino group of ornithine was modified with an acryloyl group to facilitate its attachment to the polymer chain. Additionally, N,N’-bisacryloylcystine, derived from cystine, was employed as the cross-linker. The hydrogel was obtained through a process of free radical polymerization. This hydrogel, composed only from derivatives of natural amino acids, has proven to be a competitive sorbent and has been effectively used to remove heavy metal pollutants, mainly lead, copper, and silver ions, from aqueous media. The maximum sorption capacities were ca. 155 mg·g^−1^, 90 mg·g^−1^, and 215 mg·g^−1^, respectively for Pb(II), Cu(II), and Ag(I). The material was characterized by effective regeneration, maintaining the sorption capacity at around 80%, 85%, and 90% for Cu(II), Ag(I), and Pb(II), respectively, even after five cycles. The properties of sorption materials, such as sorption kinetics and the effect of pH on sorption, as well as the influence of the concentration of the examined metal ions on the swelling ratio and morphology of the gel, were investigated. The EDS technique was employed to investigate the composition and element distribution in the dry gel samples. Additionally, IR spectroscopy was used to identify the functional groups responsible for binding the studied metal ions, providing insights into their specific interactions with the hydrogel.

## 1. Introduction

Polymeric hydrogels are a type of soft material characterized by a hydrophilic network containing an aqueous solution. These networks can be formed using a variety of natural materials, such as polysaccharides, nucleic acids, and polypeptides, as well as synthetic polymers like polyacrylamides, polyacrylates, and polyethylene glycol. They are cross-linked through both covalent and non-covalent interactions. Because of their high water content and solid-like structure, hydrogels exhibit properties that are a blend of solids and liquids. The polymer network effectively traps the solvent, reducing its fluidity. At a macroscopic scale, the three-dimensional gel network maintains the gel’s structural integrity, stores mechanical energy, and participates in deformation processes. At a microscopic level, diffusional processes of solutes occur within the gel. Hydrogels find widespread applications in various fields due to their unique properties, which encompass the capacity to absorb large amounts of water, a distinctive three-dimensional network that confers specific mechanical properties, thermal and chemical resistance, flexibility, non-toxicity, often biocompatibility, biodegradability, and the capability to adsorb heavy metal ions and organic compounds [1,2,3].

Another interesting feature of hydrogel materials, extensively explored by numerous research groups, is their capacity to undergo significant volume changes in response to changes in environmental conditions. It is known that changes in temperature, pH, or concentration of specific species/ions can lead to drastic changes in the swelling state of these materials [4]. The polymer networks of thermo-, pH-, and ion-sensitive gels contain hydrophobic moieties, ionizable groups, and functional groups capable of interacting with specific ions, respectively. Due to their sensitivity to specific ions, researchers have explored these polymeric hydrogels as potential candidates for applications such as sensors, swing absorbers, and molecular recognizers [5]. The ability to form complexes with metal ions also makes polymer hydrogels effective metal adsorbents.

Development of efficient methods for the removal of certain metal ions from solutions is of great concern due to their toxicity at relatively low concentrations and their propensity for bioaccumulation [6]. Among the various conventional approaches employed for this purpose, one of the most common methods involves the adsorption of heavy metal ions onto different solid substrates. These substrates include ion exchange resins, carbon-based sorbents, zeolites, ion chelating agents immobilized on inorganic supports, and suitably modified polymeric gels [7,8,9,10]. Furthermore, solid sorbents can be readily integrated into automated analytical procedures for the preconcentration and quantification of trace metal ions in natural waters. Consequently, there has been a growing research focus on the synthesis of novel, efficient sorbent materials.

Environmental contamination by heavy metals poses a serious threat to all living organisms and human health. Lead, copper, and silver ions are particularly toxic contaminants that can have detrimental effects when they accumulate in the body at elevated levels. Heavy metal ions are introduced into the environment through both natural processes and human activities. Lead is widely used in industries such as printing, photography, battery production, and pigment manufacturing. It is especially harmful to humans because it can replace calcium in the body, leading to its accumulation in bones and causing damage to the nervous system, kidney dysfunction, and reduced fertility. Copper and silver also represent significant environmental and health concerns. Copper is widely used in various industrial applications and can find its way into the environment through multiple routes. While copper is an essential element for living organisms in small amounts, excess exposure can be harmful. Silver, on the other hand, is utilized in various industries, including healthcare and electronics. It is known for its antimicrobial properties but can be toxic at high levels. The removal of harmful heavy metal residues from contaminated water is essential, thus creating a significant demand for efficient purification methods.

Polymer-gel sorbent materials often contain a variety of metal-ion complexing groups, including carboxyl, sulfonic, amide, pyridine, thiol, crown ether, and various other groups. In addition, the stability of the complexes formed depends on the composition of the polymer matrix and its structure. Recently, we have reported the synthesis of polymeric gels based on acryloyl derivatives of amino acids. The presence of the amino acid moieties gives the gels interesting features, such as the ability to self-heal, degradability under certain conditions, and an intriguing swelling behavior in response to changes in temperature, pH, and the concentration of metal ions [11,12,13,14,15].

The aim of this study was to synthesize a new hydrogel rich in various functional groups capable of interacting/complexing metal ions. To achieve this, acryloyl derivatives of ornithine and cystine: N-δ-acryloyl ornithine and N,N’-bisacryloylcystine were selected as constituents of the polymeric gel structure. The former introduced α-amino acid groups into the polymeric network, while the latter introduced functional groups such as disulfide, carboxylic, and amide. It was expected that this new hydrogel would serve as an effective sorbent, capable of removing heavy metal pollutants, including lead, copper, and silver ions from aqueous systems.

## 2. Results and Discussion

### 2.1. Sorption Capacity

**Figure 1:**
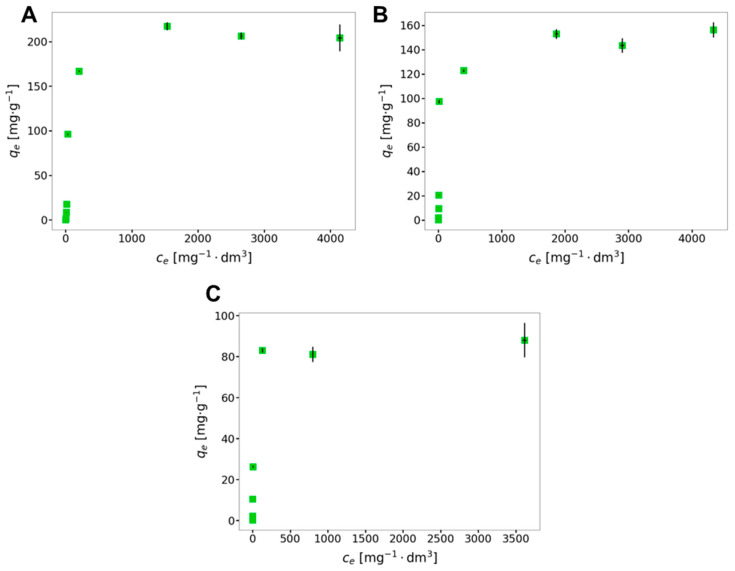
Isotherms for the sorption of Ag(I) (**A**), Pb(II) (**B**), and Cu(II) (**C**) ions by the AcOrn-BISS hydrogel. Error bars represent standard uncertainties of both correlated variables.

Sorption capacity is a crucial factor in calculating the amount of sorbent required to effectively remove pollutants, especially those present at trace levels, from the solution being tested. Figure 1 illustrates the relationship between the equilibrium concentration of metal ions and the quantity of adsorbed metal cations onto the gel. In all cases, sorption capacities increase significantly at low metal ion concentrations. At the highest concentrations, the sorption shows some variability; however, it appears to remain relatively constant. 

The results were analyzed in terms of the Langmuir, Freundlich, and Sips models. However, the Freundlich model did not fit well with the experimental data, as illustrated in the Supplementary Information in Figure S1. The Langmuir model is a two-parameter model that assumes there are no interactions between the adsorbed molecules and that only a monolayer of adsorbate can be formed. The Langmuir isotherm can be expressed in the linearized form as:

$$\frac{1}{q_{e}}=\frac{1}{K_{L}q_{m}}\cdot \frac{1}{C_{e}}+\frac{1}{q_{m}}\;$$

where *q_e_* represents the quantity of metal ions adsorbed per gram of adsorbent [mg·g^−1^], *C_e_* denotes the equilibrium concentration of the metal ions in the solution [mg·L^−1^], *q_m_* refers to the theoretical maximum adsorption capacity of the monolayer [mg·g^−1^], and *K_L_* denotes the Langmuir constant [L·mg^−1^], which reflects the affinity of the binding sites.

On the other hand, the Sips model is a three-parameter generalized isotherm model that represents a combination of the Langmuir and Freundlich models for better prediction of the adsorption in heterogeneous systems. The equation of Sips isotherm is given by the formula [16]:

$$q_{e}=\frac{q_{m}K_{s}C_{e}^{n}}{1+K_{s}C_{e}^{n}}$$

where *K_s_* denotes Sips constant [L·mg^−1^] and *n* is a fitting parameter. 

The linear form of the Sips isotherm equation resembles the Langmuir equation, but the term 1/*C_e_* raised to the power of *n*:

$$\frac{1}{q_{e}}=\frac{1}{q_{m}}+\frac{1}{q_{m}K_{s}}\cdot \frac{1}{C_{e}^{n}}$$

It becomes the Langmuir equation by setting *n* = 1.

**Figure 2:**
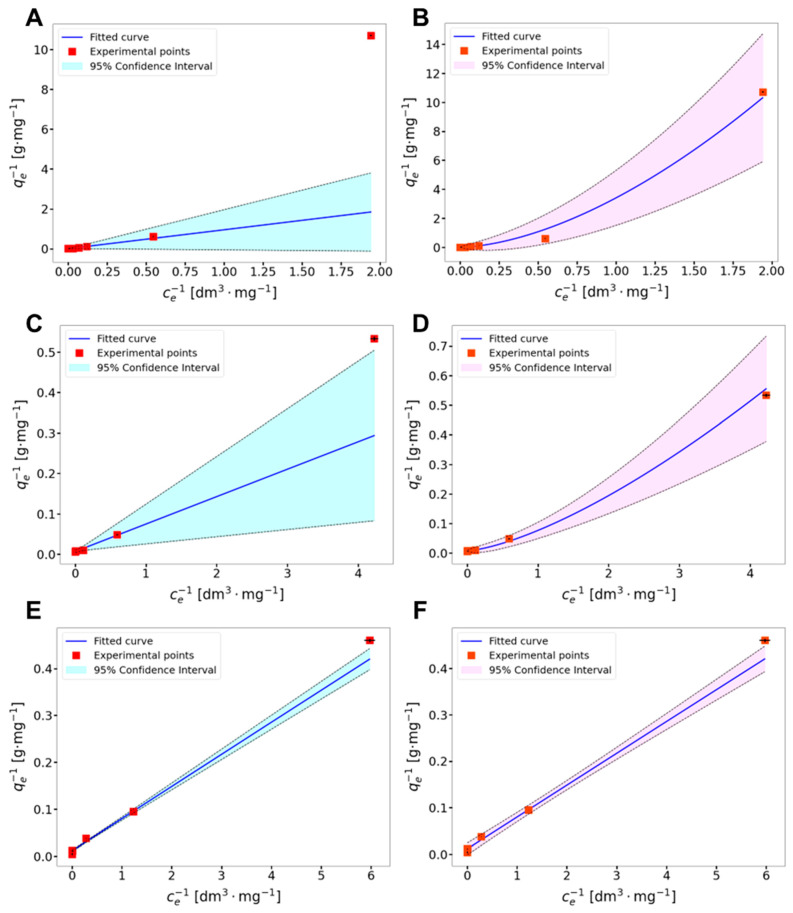
Comparison of fitting experimental data obtained from sorption experiments to Langmuir model (left column) and Sips model (right column) for: Ag(I) (**A**,**B**), Pb(II) (**C**,**D**), and Cu(II) (**E**,**F**) ions. Error bars represent standard uncertainties of both correlated variables.

To evaluate which model better depicts the sorption process of lead, copper, and silver ions with AcOrn-BISS gel, the plots of *q_e_*^−1^ vs. *c_e_*^−1^ were analyzed. In the case of the Sips isotherm model, in the first step, the weighted orthogonal distance non-linear regression method (ODR) with uncertainties in *x* and *y* was employed to find the *n* coefficient of the fitted function. The calculations were made using Python 3.11. After finding the *n* parameter, the weighted linear regression method with uncertainties in the *x* and *y* axes was used to determine the *R*^2^ coefficient [17]. The value of the *R*^2^ coefficient was used as the criterion for the selection of the most adequate isotherm model. The comparison of the obtained regression parameters is shown in Table 1. The results obtained from sorption experiments fitted to the Langmuir and Sips models are graphically presented in Figure 2. The empirical dependencies are accompanied by 95% confidence intervals (95% CIs). The calculation of the 95% CI for the regression curve is not a trivial problem, especially for the case where the regression curve is fitted by employing the weighted non-linear regression method. In weighted regression analyses in addition to residual errors, experimental uncertainties in both correlated variables are taken into account. The resulting 95% confidence bands (shown in Figure 2) are estimated by employing the rules of propagation of standard uncertainties according to the following formula:

$$CI=t(P=95\%,\;df=N-m)\cdot \sqrt{\sum_{j=1}^{m}{\left[\frac{\partial f}{\partial a_{j}}u\left(a_{j}\right)\right]}^{2}+2\sum_{j=1}^{m-1}\sum_{k=j+1}^{m}\frac{\partial f}{\partial a_{j}}\frac{\partial f}{\partial a_{k}}u\left(a_{j}\right)u\left(a_{k}\right)r_{jk}\;}$$

where *CI*—confidence interval, *t*—critical value for Student distribution at 95% confidence level, *df*—number of degrees of freedom, *N*—number of experimental points, *m*—number of regression coefficients, *f*—model function (adsorption isotherm function), and *r_jk_*—correlation coefficient for *a_j_* and *a_k_* coefficients.

It is important to emphasize that all empirical dependencies used for the prediction of the sorption isotherm model, kinetics parameters, and pH effects incorporate experimentally measured quantities affected by measurement errors. Therefore, in contrast to the vast majority of the papers that deal with sorption phenomena, in this work, modeling of the empirical dependencies via the regression analyses was performed using the weighted linear regression method that takes the combined uncertainties of the measured quantities into account. The combined uncertainties of the measured quantities are combinations of uncertainties due to the random (measurement repeatability) and systematic (instrumental) errors and are quantified by employing the rules of propagation of errors.

One can see that for the experimental data obtained for Cu(II) ions, the Sips model produces values of the fitting parameters insignificantly different from those predicted for the Langmuir model. In addition to this, the *R*^2^ coefficient is very close to one and the exponent *n* from the Sips model is also equal to one. For Pb(II) and Ag(I) ions, the exponent values from the Sips model deviate from one, which means that the sorption mechanism cannot be described by the Langmuir sorption formalism. The experimentally determined maximum sorption capacities were ca. 155 mg·g^−1^, 90 mg·g^−1^, and 215 mg·g^−1^ for Pb(II), Cu(II), and Ag(I), respectively. The comparison of these values with those of other recently described sorbents is presented in Table 2

### 2.2. Sorption Kinetics

The examination of kinetics of the sorption process for Pb(II), Cu(II), and Ag(I) ions by AcOrn-BISS gel in aqueous solutions was performed at pH = 5 and at room temperature. The analysis was based on the Weber–Morris, pseudo-first- and pseudo-second-order kinetic models. Weber–Morris model fitting to the experimental data is presented in the Supplementary Information (Figure S2).

The integral forms of equations that describe these kinetic models can be expressed as [23]:

$$\ln\left(q_{e}-q_{t}\right)=-k_{1}t+ln(q_{e})$$

for the first-order process and

$$\frac{1}{q_{e}-q_{t}}=k_{2}t+\frac{1}{q_{e}}$$

for the second-order process, where *t* represents the contact time [min], *q_t_* and *q_e_* indicate the number of ions removed at a specific time *t* and at equilibrium [mg·g^−1^], and *k*_1_ [min^−1^] and *k*_2_ [g·mg^−1^·min^−1^] are the adsorption rate constants. 

To find the most adequate kinetic model for the sorption process at the AcOrn-BISS gel system, the experimental data were fitted to the linearized kinetic equations by using the weighted linear regression method that involves uncertainties in *y* axis. The magnitude of the *R*^2^ coefficient served as the choice criterion. The obtained regression parameters are shown in Table 3.

The analysis of the regression results listed in Table 3 reveals clearly that both kinetic models may be equivalently employed for the description of the studied sorption process. The differences between the criterion *R*^2^ values for the given metal ion are insignificantly small. 

**Figure 3:**
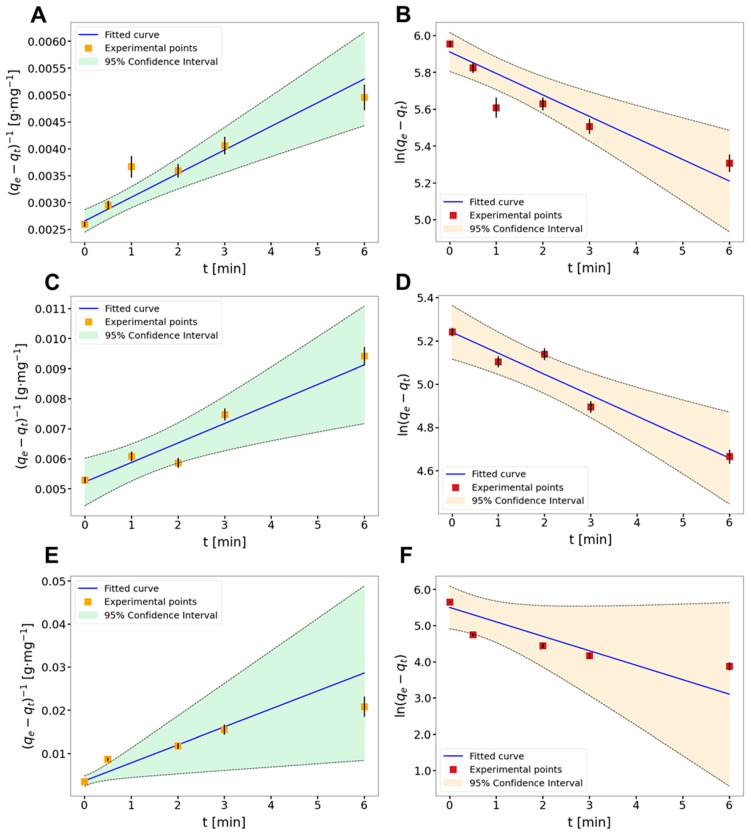
Kinetic experimental data fitted to pseudo-first-order (**right**) and pseudo-second-order (**left**) models with a confidence interval for the fitted curve. Dependence of ln(*q_e_* − *q_t_*) (pseudo-first-order model) and inverse of the (*q_e_* − *q_t_*) (pseudo-second-order model) vs. time *t* for Ag(I) (**A**,**B**), Pb(II) (**C**,**D**), and Cu(II) (**E**,**F**) ions. Error bars represent standard uncertainties of dependent variables.

The regression lines for the pseudo-first- and pseudo-second-order models are illustrated in Figure 3.

### 2.3. Effect of pH

**Figure 4:**
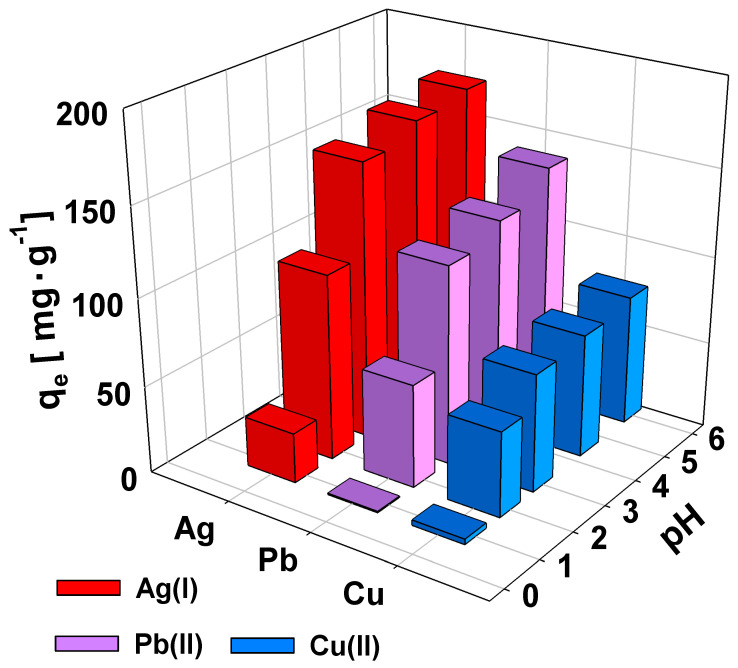
Effect of solution pH on the sorption of Pb(II), Cu(II), and Ag(I) ions by AcOrn-BISS gel. Initial metal ions concentrations: 1000 mg·L^−1^, room temperature.

The pH level of the solution plays a crucial role in determining the efficiency of heavy metal ion adsorption. It influences the sorbent by either protonating its functional groups or causing the metal ions to form metal oxides or hydroxides. To explore how pH affects the adsorption of the metal ions under study, experiments were carried out within a pH range of 1 to 6 to avoid the risk of metal ion precipitation. As shown in Figure 4, the sorption capacities for Cu(II), Pb(II), and Ag(I) increase with an increase in pH. At pH = 1, the sorption percentage was the lowest, probably because of the extensive protonation of the functional groups in the polymer network. As the pH increased, the polymer network became more negatively charged due to the ionization of acidic groups. This enhanced the formation of complexes between the polymer and metal ions, leading to improved sorption efficiency. Notably, sorption efficiency remained high across the pH range from 3 to 6.

### 2.4. Regeneration of Hydrogel Sorbent

**Figure 5:**
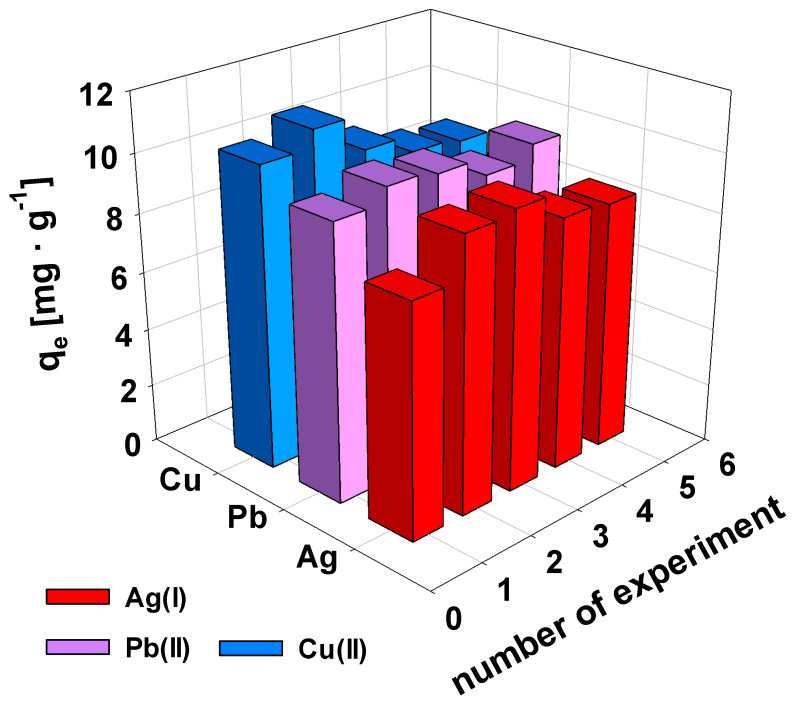
Efficiency of regeneration of sorption material using EDTA measured by sorption capacity of AcOrn-BISS gel for repeated sorption cycle for Ag(I), Cu(II), and Pb(II) cations present at a fixed initial concentration of 50 mg·L^−1^.

The regeneration of sorbents is an important aspect from both an economic and environmental point of view. The possibility of regenerating AcOrn-BISS gel using HNO_3_ or EDTA was examined. It was observed that the employment of HNO_3_ makes the sorption capacity decrease gradually, reaching ca. 20% after three cycles. In contrast, the employment of EDTA led to more promising results. The sorption capacity for all tested ions only experienced a minor decrease, remaining at around 80%, 85%, and 90% for Cu(II), Ag(I), and Pb(II), respectively, even after five cycles (see Figure 5).

### 2.5. Swelling Behavior of Hydrogel Sorbent after Metal Absorption

**Figure 6:**
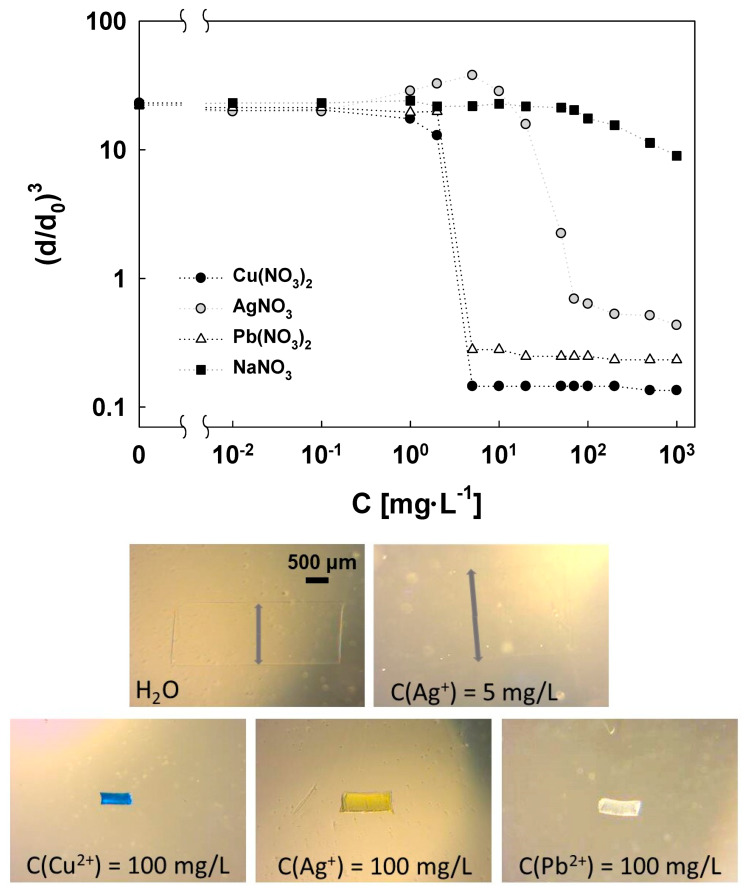
Influence of Cu(II), Pb(II), and Ag(I) ion concentrations on the swelling ratio of the AcOrn-BISS gel at T = 20 °C and photos of the gel samples for the selected metal ion concentration. The scale presented in the photograph in pure water is applied to all photographs.

In the next stage of hydrogel characterization, the influence of Cu(II), Pb(II), and Ag(I) ion concentrations on the swelling ratio of the AcOrn-BISS gel was examined. The relation between the swelling ratio, defined as ${\left(\frac{d}{d_{o}}\right)}^{3}$, and the concentration of the tested ions is presented in Figure 6. In the case of Cu(II) and Pb(II) ions, the dependences have a sigmoidal shape, and the swelling ratios decrease with an increase in the concentration of the metal ions. The gel exhibits the highest sensitivity to these ions when they are present at concentrations in the range of 1-10 mg·mL^−1^. This behavior can be explained in terms of the interaction between metal ions and the functional groups within the polymeric network of the AcOrn-BISS gel, involving various stoichiometries. It is known that amino acids can form stable complexes with certain metal cations. Typically, two types of complexes with stoichiometry of 1:1 and 1:2 can be formed [24,25]. In the case of the formation of 1:1 complexes, the expansion of the polymer network, due to the introduction of an excess positive charge into the polymeric chains, is expected. Copper(II) and lead(II) ions form complexes with glycine of relatively high stability constants. The values of the stability constants for the complexes with glycine are the following: $\log\beta_{ML}$~8 and $\log\beta_{{ML}_{2}}$~15 for Cu(II) and $\log\beta_{ML}$~5 and $\log\beta_{{ML}_{2}}$~9 for Pb(II), respectively [25]. It was also demonstrated that Cu(II) can form complexes with cystine in the aqueous medium with stoichiometry 1:1 and 1:2 [26].

Silver ions exhibit a different behavior. Interestingly, an increase in Ag(I) concentration in the range from 1 mg·L^−1^ to 10 mg·L^−1^ initially results in an increase in the swelling ratio, followed by a significant shrinkage of the gel in the range of 10 mg·L^−1^ to 100 mg·L^−1^. Among the investigated cations, Ag(I) formed complexes with glycine with the lowest stability constant [26]. Also, silver ions interact rather weakly with carboxylic groups and more effectively with disulfide bonds from BISS. This behavior can be explained by the dominance of complexes with a stoichiometry of 1:1 between Ag(I) and the polymer network in the lower concentration range (up to 10 mg·L^−1^), while the shrinking process at higher concentrations is caused by the formation of complexes with a stoichiometry of 1:2.

For comparison purposes, the effect of sodium cation concentration on swelling ratio was also investigated. In this case, only for concentrations above 100 mg·L^−1^, a slight effect on the swelling ratio is apparent. It can be assumed that sodium cations do not interact with the polymeric network, and at high concentrations, the so-called “salt effect” leads to a decrease in the swelling ratio.

In Figure 6, photographs of the cylindrical gel samples before and after adding metal ions are presented. For this purpose, the concentration of metal ions of 100 mg·L^−1^, at which the gels are characterized by the smallest swelling ratio, was selected. As can be observed, in addition to the significant change in volume, the gel samples also displayed distinctive colors: blue for Cu(II), yellow for Ag(I), and white for Pb(II). A photo of the gel sample is also provided at a concentration of 5 mg·L^−1^ of Ag(I) when the swelling ratio was at its peak. In this case, the increase in the swelling ratio made the boundary of the cylindrical gel sample hardly visible, and the diameter was indicated with a gray arrow.

As the quantity of adsorbed ions increases, the polymer network contracts, which improves the mechanical properties of the hydrogel and makes it easier to remove the sorbent from the solution.

### 2.6. FTIR Spectroscopic Analysis

**Figure 7:**
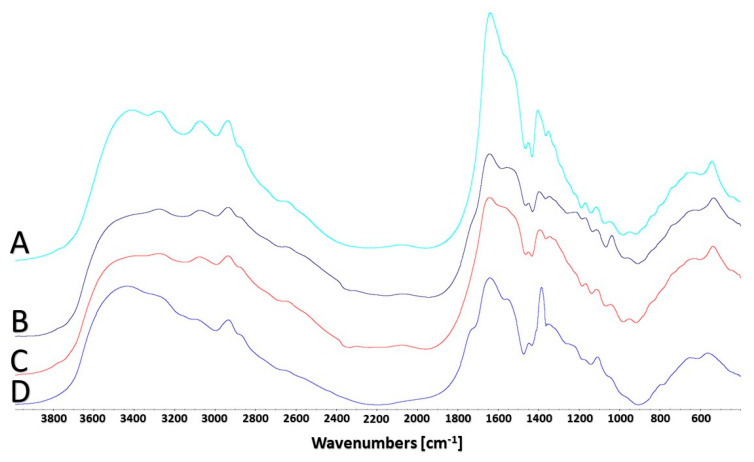
FTIR spectra of dried samples of NIPS-BISS hydrogels before (A) and after adsorption of silver (B), lead (C), and Cu copper (D) ions.

FTIR spectroscopy was employed to explain the possible mechanism of adsorption of Ag(I), Pb(II), and Cu(II) ions in the AcOrn-BISS gel. To investigate the role of functional groups in the sorbent involved in ion binding, FTIR spectra were recorded before (Figure 7A) and after the adsorption of Ag(I), Pb(II), and Cu(II) ions (see Figure 7B–D). Before the adsorption process, two broad bands located at 3410 and 3280 cm^−1^ are clearly visible. These signals can be assigned to the stretching of the N-H bonds of the amine and amide groups from the AcOrn and BISS components, respectively. After the adsorption process, these bands are perturbed. The bands in a range of 3100–2800 cm^−1^, that are insensitive to the sorption process, can be assigned to the asymmetric and symmetric vibrations of the methyl and methylene groups. Next, a weak signal that can be assigned to the –OH group from carboxylic groups appeared at 2660 cm^−1^. As can be seen, this signal is affected by the sorption process. At 1640 cm^−1^ the highest signal is located. This signal is characteristic of C=O bonds from amide groups. The position of this signal is not affected by the sorption process. Next, a signal at 1550 cm^−1^ can be assigned to asymmetric stretching of the -COO^-^ groups. This signal is strongly affected by the sorption process. At approximately 1700 cm^−1^, an overlapped signal characteristic of C=O bonds from –COOH groups is observed, suggesting that a certain portion of the carboxyl groups is protonated, which is also confirmed by a weak signal located at 2660 cm^−1^. Based on the changes observed in the FTIR spectra after the sorption process, it can be concluded that the amine, amide, and carboxylic groups from the AcOrn and BISS components of the sorbent make the predominant contribution to the sorption of Ag(I), Cu(II), and Pb(II) ions. It is worth noting that determining the interactions of metal ions with –S–S– groups is challenging because these groups give rise to weak signals in the fingerprint region and are usually masked by stronger bands from other functional groups. However, it is well known that disulfide bonds can interact with metal ions through the sulfur atoms, which have lone pairs of electrons that can participate in coordination bonding [27]. This interaction can enhance the stability of metal ion binding, especially with Ag(I), which is a “soft” Lewis acid, because –S–S– groups are “soft” Lewis bases. Additionally, interactions with Cu(II) and Pb(II), which are borderline Lewis acids, cannot be neglected.

### 2.7. Characterization of Sorbent Morphology and Element Distribution 

**Figure 8:**
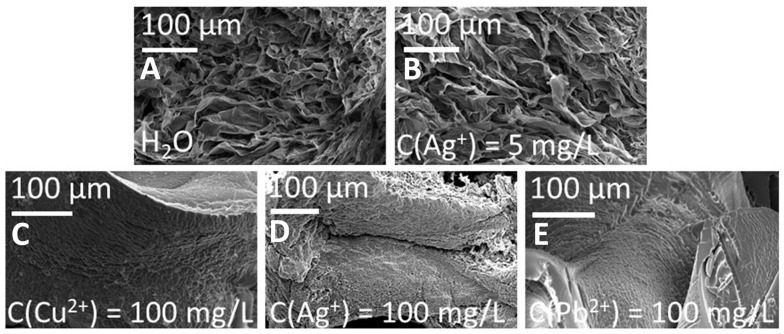
SEM images of lyophilized AcOrn-BISS hydrogel samples before (**A**) and after the sorption of Ag(I) (**B**,**D**), Cu(II) (**C**), and Pb(II) (**E**) ions from solutions with selected metal ion concentrations.

The morphology of lyophilized samples of AcOrn-BISS hydrogels conditioned in water and in solution of metal cations are visualized by SEM images presented in Figure 8. The concentration of metal ions was the same as in the sample where photos in the solution were taken. Before sorption (swollen in water) the gel presents a highly porous structure. As expected, the gel conditioned in a 5 mg·L^−1^ Ag(I) solution also reveals a highly porous structure, but the pore diameter appeared to be larger. After treating the hydrogel with solutions containing the investigated metal ions at a concentration of 100 mg·L^−1^, significant changes in morphology were observed. The structure became denser, and pores appeared to be much smaller. Only in the case of Ag(I), the porous structure is still clearly visible, while for Cu(II) and Pb(II), the polymer structure appears to be more solid. These observations are in good agreement with the results of the swelling behavior experiments.

**Figure 9:**
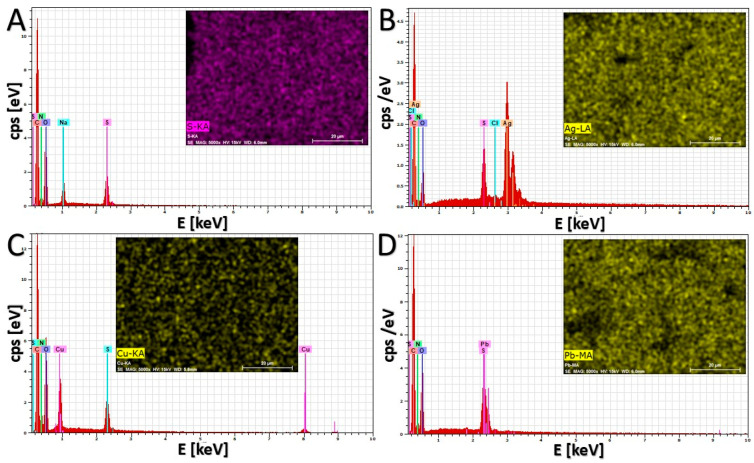
EDS spectra and selected elements distribution for dried AcOrn-BISS hydrogel samples before (**A**) and after the sorption of silver (**B**), copper (**C**), and lead (**D**) ions.

The EDS technique was employed to investigate the composition and element distribution in the dry gel samples. The obtained results are presented in Figure 9. As can be seen, signals from the elements that form the polymer network of the gel (C, N, O, and S) are clearly visible in all spectra. Additionally, for the gel after the sorption process, characteristic signals for the metal are present. The distribution of characteristic elements such as S, Ag, Cu, and Pb in the gel samples before and after sorption is uniform.

The oxidation state of metal ions within the AcOrn-BISS hydrogel samples was investigated using X-ray photoelectron spectroscopy (XPS). XPS spectra recorded for the gel sample after adsorption of Cu(II) showed characteristic signals for O 1s, C 1s, N 1s, S 2p, and Cu 2p (see Figure S3). The presence and localization of Cu 2p signals confirm the +2 oxidation state of copper ions [28]. For the gel after Pb(II) sorption, XPS spectra (see Figure S4) displayed signals for O 1s, C 1s, N 1s, S 2p, and Pb 4f. The position of the Pb 4f signals suggests a +2 oxidation state of lead ions [29]. Additionally, it is not expected that Pb(II) would be oxidized within the AcOrn-BISS gel. Silver ions (Ag(I)) are particularly susceptible to changes in oxidation state, and reduction to Ag(0) could be anticipated. However, based on the literature, distinguishing between Ag(I) and Ag(0) using XPS is generally not feasible [30]. Nonetheless, after several weeks of storage, samples containing Ag(I) exhibited dark discoloration on the surface, suggesting reduction. Upon breaking the sample, the color was a light yellow, which indicates that the reduction was likely limited to the surface layer.

## 3. Conclusions

A novel synthesis route for crosslinked polymeric gels based on acryloyl derivatives of natural α-amino acids, ornithine, and cystine, was carefully designed and implemented for tests in a laboratory scale. The prepared hydrogel (acryloyl N-δ-acryloyl ornithine and N,N’-bisacryloyl cysteine—AcOrn-BISS gel) is rich in a variety of functional groups capable of complexing metal ions, and, thus, it is a very promising sorbent material for the efficient removal of metal ions from the aqueous system. The studies were performed for three selected metal ions, i.e., Cu(II), Pb(II), and Ag(I), which are crucial for electronic-waste materials processing toward the recovery of metallic materials. The fundamental sorption characteristics revealed the great potential of AcOrn-BISS gel for metal accumulation, its efficient regeneration (especially in an EDTA environment) in a number of repeated sorption cycles, and resistance to the changes in the solution pH down to 2.5. The experimentally determined maximum sorption capacities at pH = 5 were ca. 155 mg·g^−1^, 90 mg·g^−1^, and 215 mg·g^−1^ for Pb(II), Cu(II), and Ag(I), respectively. The Sips rather than Langmuir model turned out to be the best formalism describing the sorption process of the selected metal ions by AcOrn-BISS hydrogel. The sorption mechanism was explained in terms of the interaction between metal ions and the functional groups within the polymeric network of the AcOrn-BISS gel, involving formation of complexes of either 1:1 or 1:2 stoichiometries. Consequently, an increase in the number of adsorbed ions enhances the process of shrinking the polymer network and, as a result, facilitates the removal of the sorbent from the solution. All these effects were evidenced by using optical and scanning electron microscopies. The EDS technique revealed a uniform distribution of metal ions and sulfur throughout the dry gel samples, demonstrating even incorporation of these elements. FTIR spectra indicated that the amine, amide, and carboxylic groups from the AcOrn and BISS components of the sorbent make a predominant contribution to the sorption of Ag(I), Cu(II), and Pb(II) ions.

## 4. Materials and Methods 

### 4.1. Materials 

L-ornithine monohydrochloride salt (99%), L-cystine (98.5%), acryloyl chloride (96%), copper(II) nitrate trihydrate (98%), N,N,N′,N′-tetramethylethylenediamine (TEMED), and ammonium persulfate (APS) were purchased from Sigma-Aldrich, Poznań, Poland. Sodium hydroxide (NaOH, 99%) and hydrochloric acid (HCl, 35–38%) were purchased from POCh. Lead(II) nitrate (≥99%) and silver nitrate (99%) were purchased from Honeywell. Ultrapure nitric acid (67%) was purchased from Merck, Poznań, Poland.

All chemicals were utilized in their received form without any further purification. Solutions were prepared using high-purity water obtained from a Milli-Q Plus/Millipore purification system, Poznań, Poland, ensuring a water conductivity of 0.056 μS·cm^−1^. The N-δ-acryloyl ornithine monomer (AcOrn) and the N,N’-bisacryloylcystine cross-linker (BISS) were synthesized following established methods described in previous research [11]. 

In the context of N-δ-acryloyl ornithine, the procedure involved adding ornithine monohydrochloride to a NaOH solution. A solution with CuSO_4_·5H_2_O was introduced, resulting in a deeply blue solution, which was then cooled to 10 °C. Acryloyl chloride and NaOH were gradually added dropwise while maintaining the pH between 9 and 10. After completion, the reaction mixture was stirred overnight at room temperature. The blue precipitate formed was filtered, washed, and dried, resulting in the N-δ-acryloyl ornithine-copper complex. Thioacetamide was introduced to a powdered suspension of the complex in water, stirred for 20 min, and the pH was adjusted to 9. This led to the formation of a copper sulfide precipitate, which was filtered, yielding a colorless filtrate. After evaporating the water, a residue was dissolved in a mixture of MeOH and CF_3_COOH. Et_2_O was used for precipitation, and the crude product underwent recrystallization with MeOH and Et_2_O.

To synthesize N,N’-bisacryloylcystine, the following procedure was utilized: a solution containing sodium hydroxide and cystine in methanol underwent stirring, and acryloyl chloride was cautiously added dropwise at 0 °C. The resultant solution was further stirred at ambient temperature. After a duration of approximately 4 h, the reaction mixture was subjected to filtration using a celite pad. The filtrate was then gradually introduced dropwise into vigorously stirred cold diethyl ether. The resulting suspended solid was separated through filtration, treated with diethyl ether washing, and subsequently dried using high vacuum conditions within the range of 30–45 °C. The analysis based on sulfur content from combustion analysis revealed the presence of approximately 65% of the disodium salt of N,N′-bisacryloylcystine in the powder.

The successful synthesis of N-δ-acryloyl ornithine and N,N’-bisacryloyl cysteine was confirmed using ^1^H NMR, ^13^C NMR, and mass spectroscopy techniques.

### 4.2. Synthesis of AcOrn-BISS Hydrogel

**Figure 10:**
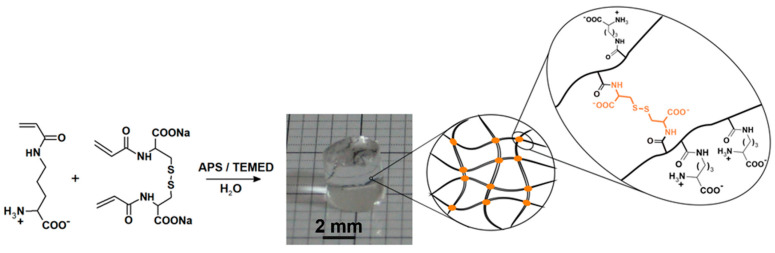
Scheme of synthesis of the AcOrn-BISS hydrogel.

AcOrn-BISS gel was synthesized through a free-radical solution copolymerization. The total concentration of AcOrn and BISS was 560 mM, with the AcOrn component accounting for 80% of this concentration. To prepare the pre-gel solutions, deoxygenation was conducted. Subsequently, initiation and acceleration were achieved by introducing APS (1.88 mM) and TEMED (32 mM) at a temperature of 5 °C for 20 h. After the polymerization process, the resulting gels were soaked in deionized water for a week to remove any residual impurities. The water was regularly changed throughout this washing period to ensure thorough cleaning. Subsequently, the synthesized gels were dried at a temperature of 50 °C and then ground into a fine powder. Figure 10 illustrates a schematic diagram of the synthesis process for the AcOrn-BISS hydrogel.

### 4.3. Evaluating the Sorption Properties

#### 4.3.1. Adsorption Isotherm

To assess the sorption capacity, 20 mg of the dried polymer samples was mixed with 4 mL of a metal-ion solution. The concentration range was 1–5000 mg/L for Pb(II), Ag(I), and Cu(II). The mixtures were shaken for 24 h at room temperature followed by decantation. Then, the samples were filtered using cotton wool and a syringe. The residual amounts of metal ions in the tested solutions were determined via inductively coupled plasma optical emission spectroscopy (ICP-OES, a Perkin Elmer Avio 200 model, PerkinElmer, Kraków, Poland). The quantification results used for the construction of the experimental dependencies were averaged from at least 3 replicate measurements.

The sorption capacity, denoted as *q_e_* [mg·g^−1^], was calculated using the formula: $q_{e}=\frac{\left[\left(C_{0}-C_{e}\right)V\right]}{m}$. In this equation, *C*_0_ and *C*_e_ represent the initial and equilibrium concentrations [mg/L] of the metal ion in the aqueous solution, respectively. V is the volume of the metal-ion solution in liters [L], and mm is the mass of the sorbent in grams [g].

#### 4.3.2. Adsorption Kinetics

A 60 mL aqueous sample of either Cu (II) (3000 mg·L^−1^) or Ag (I) (3500 mg·L^−1^) or Pb (II) (2500 mg·L^−1^) was prepared in glass bottles with stoppers. The pH value of the prepared samples was around 5. A portion of 300 mg of the adsorbing material was inserted into a small synthetic nonwoven fabric bag and then the system was transferred into each bottle. The bags were tested prior to the kinetic tests and showed no adsorption properties toward examined ions. The systems were stirred for 24 h at room temperature on a magnetic stirrer. The sorption capacities for copper, silver, and lead ions were calculated by measuring the difference between the initial concentrations and the equilibrium concentrations of Cu(II), Ag(I), and Pb(II) ions. During kinetic studies, samples were taken from the solution phase at various time intervals. The concentrations of Cu(II), Ag(I), and Pb(II) ions were analyzed using ICP-OES. The quantification results used for the construction of the experimental dependencies were averaged from at least 3 replicate measurements.

#### 4.3.3. pH Dependence Study

A portion of 25 mg of the adsorbing materials was added to a 5 mL aqueous sample of Cu(II) (1000 mg·L^−1^), Ag(I) (1000 mg·L^−1^), and Pb(II) (1000 mg·L^−1^). The pH values of the samples were adjusted with HNO_3_ solution. Sorption capacities were tested in the pH range from 1 to 6. The systems were gently shaken for 24 h at room temperature. Then, the samples were filtered using cotton wool and a syringe and the concentrations of Cu(II), Ag(I), and Pb(II) were determined by ICP-OES. Sorption capacities were calculated using the formula presented in the “Adsorption isotherm” section.

#### 4.3.4. Metal Recovery Experiments

A 20 mL aqueous sample of Cu(II), Ag(I), or Pb(II) of known concentration was prepared in glass bottles with stoppers. Concentrations of metal ions were 50 mg·L^−1^ for metal recovery in EDTA experiments and 1000 mg·L^−1^ for recovery examination in nitric acid. A portion of 100 mg of the adsorbing material was inserted into a nonwoven fabric bag and then the system was transferred into each bottle. The prepared systems were stirred for 24 h at room temperature. Later on, the samples were filtered using cotton wool and a syringe. The concentrations of Cu(II), Ag(I), and Pb(II) were determined by ICP-OES, and sorption capacities were determined using the formula given in the “Adsorption isotherm” section. After the experiments, the bags with adsorbing material were transferred into 20 mL solutions of either EDTA or HNO_3_ (pH = 1) and stirred for another 24 h. Then, the adsorbing materials were dried and used in the next cycle of experiments. This procedure was repeated 5 times for EDTA and 3 times for HNO_3_.

### 4.4. Measurement of Swelling Ratio of Gel Rods

The changes in gel volume due to varying metal ion concentrations were assessed by measuring the diameters of rod-shaped gel samples. This was accomplished using an inverted optical microscope (Olympus, model PME 3, Tokyo, Japan) equipped with a calibrated scale. A refrigerated circulator (Polystat, Cole Parmer, Sunbury-on-Thames, United Kingdom) was used to keep the temperature constant at 20 °C during the measurements.

The swelling ratio of the cylindrical gel samples is given by the equation $\frac{V}{V_{o}}=\left(\frac{l}{l_{o}}\right){\left(\frac{d}{d_{o}}\right)}^{2}$, where *V* and *V_o_* represent the equilibrium and initial volumes of the gel, respectively. In this context, *d* is the diameter, *l* is the length of the hydrogel rod, and *d_o_* and *l_o_* are the respective diameter and length of the capillary in which the gel was synthesized. Due to significant uncertainty in accurately measuring the length of the gel rods, because their ends are often not well-defined, a simplified equation $\frac{V}{V_{o}}\;\approx \;{\left(\frac{d}{d_{o}}\right)}^{3}$ was used. The precision of the diameter measurements of the gel rods was within 5%. For this study, *d_o_* was 500 μm.

### 4.5. Scanning Electron Microscope (SEM) Investigations

The surface morphology of the lyophilized gel samples and element distribution were visualized using a Merlin, ZEISS Field Emission Scanning Electron Microscope (FE-SEM, Jena, Germany). This microscope was coupled with a Quantax 400, Bruker EDS/EDX detector, Billerica, MA, USA. To capture the morphology, the samples were freeze-dried. The samples were first frozen in liquid nitrogen to maintain the porous structure of the gels, and then lyophilized using a Labconco FreeZone Lyophilizer at a temperature of −82 °C and a vacuum of 0.03 mbar. Finally, the samples were sputtered with a palladium layer. Prior to SEM analysis, an ultra-thin film of Au-Pd alloy was applied to the samples to prevent electrical charging of the surfaces. This alloy layer, with an average thickness of 1–2 nm, was deposited using a Polaron SC7620 Mini Sputter Coater, London, United Kingdom).

### 4.6. X-ray Photoelectron Spectroscopy (XPS)

XPS measurements were performed with a Kratos Axis Supra spectrometer (Kratos Analytical Ltd., Manchester, United Kingdom) equipped with a monochromatic Al Kαradiation source (1486.7 eV). All data were collected with an analyzer with pass energy of 80 eV for the survey scan and 20 eV for region spectra, respectively. The instrument work function was calibrated to give a BE of 84.0 ± 0.1 eV for the 4f7/2 line of metallic gold and the spectrometer dispersion was adjusted to give a BE of 932.6 eV for the Cu 2p3/2 line of metallic copper. The effect of sample charging was reduced by a co-axial neutralization system. The occurring shift in energy scale was corrected by setting the main component of C1s at the literature value of 284.8 eV for adventitious carbon [31]. Peak fitting of the data was performed with the use of a Shirley background type. A convolution of GL(30) line shapes was used to fit the individual peaks.

## Figures and Tables

**Table 1:** Comparison of slope *a*, intercept *b*, *n* coefficient (only Sips model), and Pearson coefficients (*R*^2^) for linearized Langmuir and Sips isotherm models.

Metal Ion	Parameter	Langmuir Model	Sips Model
Ag(I)	*a*	0.95 ± 0.43	3.44 ± 0.41
	*b*	0.0014 ± 0.0037	0.00534 ± 0.00071
	*n*	–	1.69107524
	*R* ^2^	0.43	0.91
Cu(II)	*a*	0.0684 ± 0.0016	0.0684 ± 0.0016
	*b*	0.01154 ± 0.00080	0.01154 ± 0.00080
	*n*	–	1.0003975
	*R* ^2^	0.997	0.997
Pb(II)	*a*	0.068 ± 0.019	0.0688 ± 0.0072
	*b*	0.0069 ± 0.0013	0.00772 ± 0.00032
	*n*	–	1.43989721
	*R* ^2^	0.65	0.95

**Table 2:** Comparison of silver, copper, and lead ion-sorption capacities of selected sorbents.

Sorbent	Sorption Capacity (mg g^−1^)			Reference
	Cu(II)	Pb(II)	Ag(I)	
Activated charcoal from *Azadirachta indica*	185.8	205.6	–	[18]
H_3_PO_4_ dehydrated carbon	–	41.5	312.5	[19]
Multiwalled carbon nanotubes	24.5	97	–	[20]
MnO_2_ intercalated with H^+^ or K^+^	–	517	–	[21]
Chitosan modified with 2-mercaptobenzimidazole	–	–	350	[22]
AcOrn-BISS gel	90	155	215	This work

**Table 3:** Regression coefficients (slope *a*, intercept *b,* and Pearson correlation coefficients (*R*^2^)) of fitting the experimental data (obtained for Ag(I), Cu(II), and Pb(II) ions) to the pseudo-first- and pseudo-second-order kinetic models by using weighted linear regression.

Metal Ion	Parameter	Pseudo-First-Order Kinetic Model	Pseudo-Second-Order Kinetic Model
Ag(I)	*a*	−0.117 ± 0.019	0.000440 ± 0.000057
	*b*	5.911 ± 0.038	0.002656 ± 0.000076
	*R* ^2^	0.90	0.94
Cu(II)	*a*	−0.40 ± 0.14	0.0042 ± 0.0011
	*b*	5.50 ± 0.19	0.00363 ± 0.00037
	*R* ^2^	0.72	0.84
Pb(II)	*a*	−0.097 ± 0.015	0.00065 ± 0.00012
	*b*	5.240 ± 0.039	0.00522 ± 0.00025
	*R* ^2^	0.94	0.90

## Data Availability

Data are contained within the article and Supplementary Materials.

## Supplementary Materials

The following supporting information can be downloaded at: https://www.mdpi.com/article/10.3390/gels10090560/s1, Figure S1: Fitting experimental data obtained from sorption experiments to Freundlich model for: Ag(I) (A), Pb(II) (B) and Cu(II) (C) ions. Error bars represent standard uncertainties of both correlated variables; Figure S2: Fitting experimental data obtained from sorption kinetic experiments to Weber–Morris model: Ag(I) (A), Pb(II) (B) and Cu(II) (C) ions. Error bars represent standard uncertainties of both correlated variables; Figure S3: XPS spectra for AcOrn-BISS hydrogel samples after the sorption of Cu(II) ions. Figure S4: XPS spectra for AcOrn-BISS hydrogel samples after the sorption of Pb(II) ions.
