# Supplementary material for: Smart Hydrogel Based on Derivatives of Natural α-Amino Acids for Efficient Removal of Metal Ions from Wastewater
Source: Gels. 2024 Aug 29;10(9):560. doi: 10.3390/gels10090560 (PMC11431252; doi:10.3390/gels10090560)
Supplement: Supplementary file 1 [file gels-10-00560-s001.zip › gels-3139503-supplementary.pdf]

Supplementary Materials

Smart Hydrogel Based on Derivatives of Natural α-Amino Acids

for Efficient Removal of Metal Ions fromWastewater

Sorption isotherm

The Freundlich model features a heterogeneous system. In this model 1/n is a heterogeneity factor. This model describes also a reversible adsorption. It is not limited to the formation of the monolayer. The Freundlich isotherm is given in linearized form as:

$$\ln q_e = \frac{1}{n} \ln C_e + \ln K_F$$

where K_F (mg/g)(L/mg)$^{1/n}$ and n (dimensionless) are constants depicting the adsorption process.

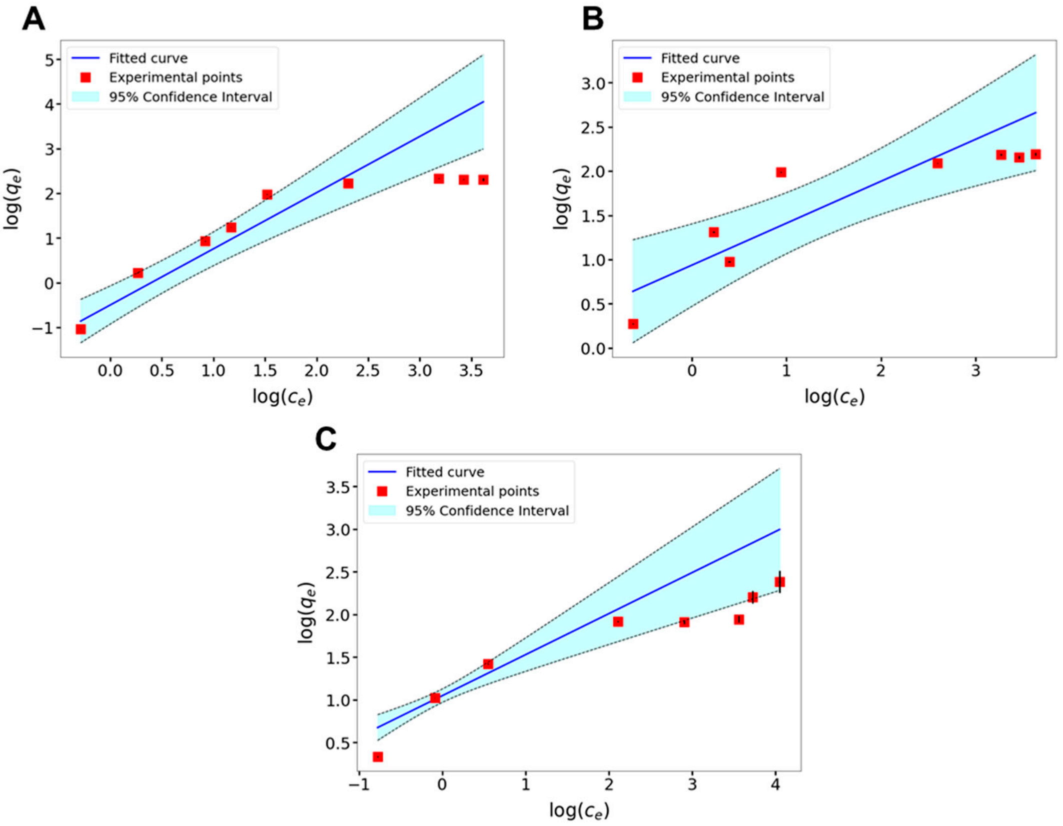

Figure S1. Fitting experimental data obtained from sorption experiments to Freudlich model for: Ag(I) (A), Pb(II) (B) and Cu(II) (C) ions. Error bars represent standard uncertainties of both correlated variables.

Sorption kinetics

The Weber–Morris model assumes that, in many adsorption scenarios, the solute uptake is nearly proportional to $t^{1/2}$ rather than to the contact time t:

$$q_t = K_{int} t^{1/2}$$

In this model, K_{int} represents the intraparticle diffusion rate constant. When intraparticle diffusion is the rate-limiting step, a plot of qt versus $t^{1/2}$ should yield a straight line with a slope of K_{int}. The straight line should pass through the origin if intraparticle diffusion is the sole mechanism for adsorption.

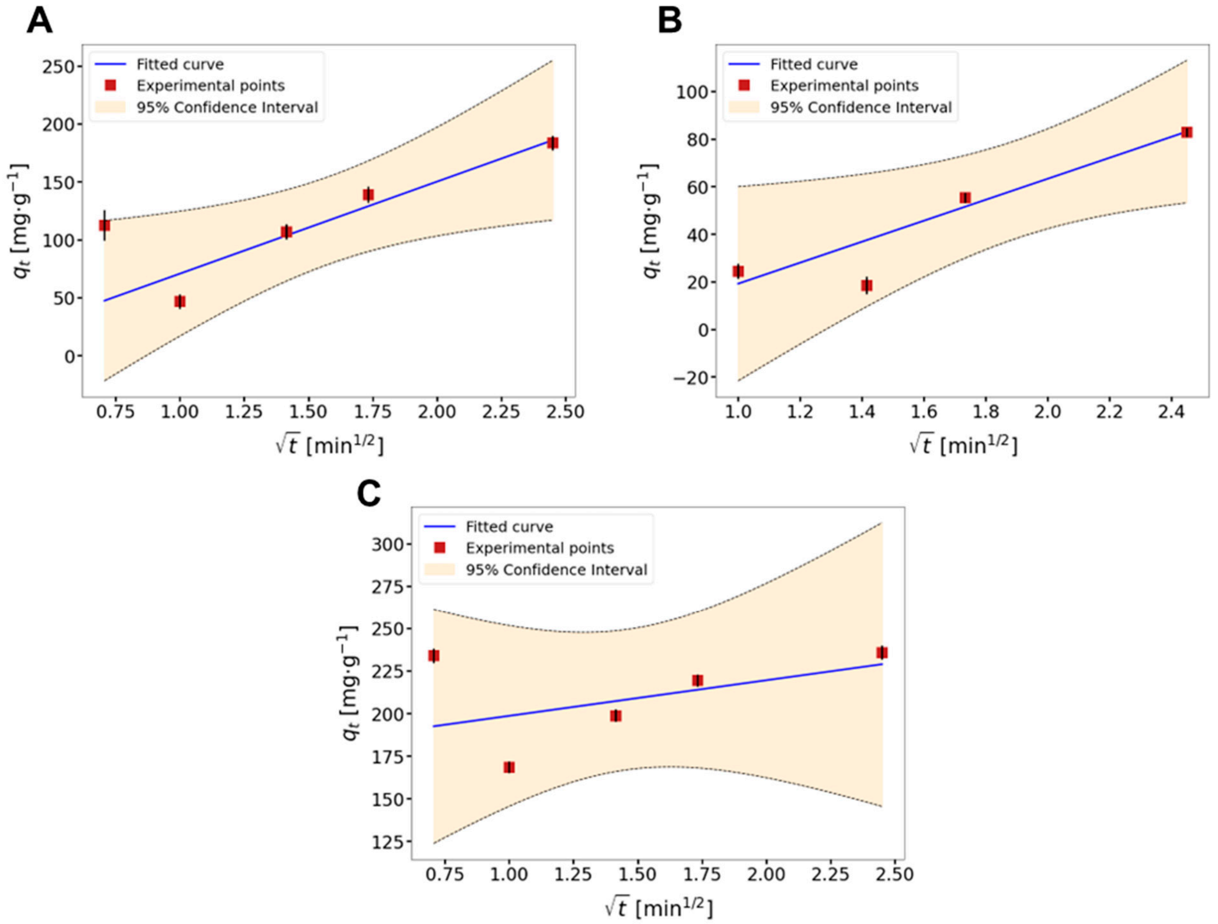

Figure S2. Fitting experimental data obtained from sorption kinetic experiments to Weber–Morris model: Ag(I) (A), Pb(II) (B) and Cu(II) (C) ions. Error bars represent standard uncertainties of both correlated variables.

X-ray photoelectron spectroscopy (XPS) investigation

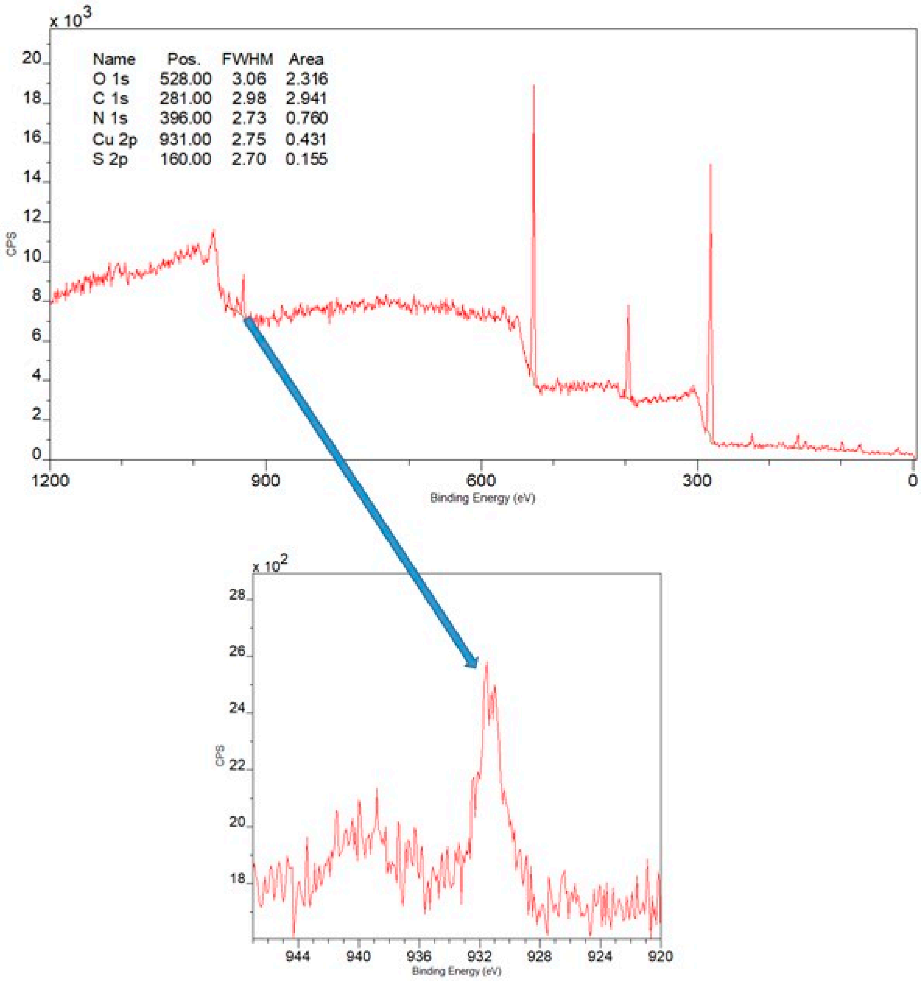

Figure S3. XPS spectra for AcOrn-BISS hydrogel samples after the sorption of Cu(II) ions

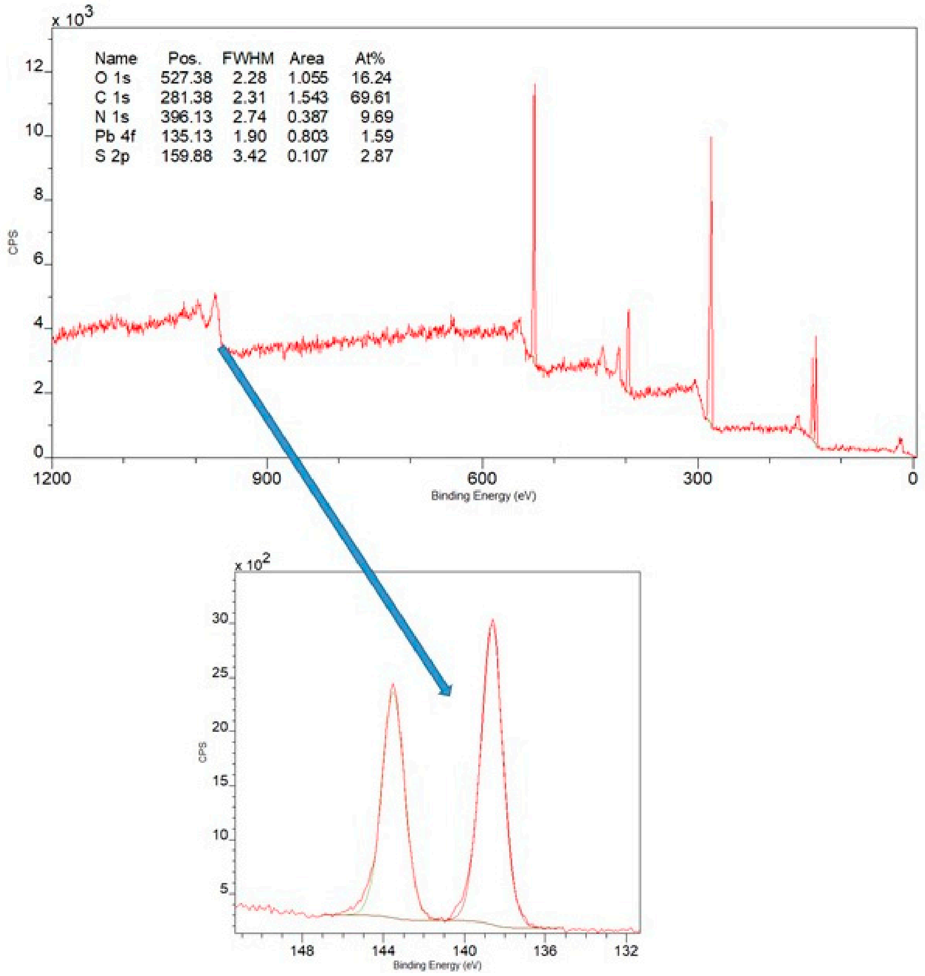

Figure S4. XPS spectra for AcOrn-BISS hydrogel samples after the sorption of Pb(II) ions.
